# The Drug-Loaded Electrospun Poly(ε-Caprolactone) Mats for Therapeutic Application

**DOI:** 10.3390/nano11040922

**Published:** 2021-04-04

**Authors:** Alena Opálková Šišková, Mária Bučková, Zuzana Kroneková, Angela Kleinová, Štefan Nagy, Joanna Rydz, Andrej Opálek, Monika Sláviková, Anita Eckstein Andicsová

**Affiliations:** 1 Polymer Institute of Slovak Academy of Sciences, Dúbravská Cesta 9, 845 41 Bratislava, Slovakia; zuzana.kronekova@savba.sk (Z.K.); angela.kleinova@savba.sk (A.K.); 2 Institute of Molecular Biology, Slovak Academy of Sciences, Dúbravská Cesta 9, 845 51 Bratislava, Slovakia; maria.buckova@savba.sk; 3 Institute of Materials and Machine Mechanics, Slovak Academy of Sciences, Dúbravská Cesta 9, 845 13 Bratislava, Slovakia; nagy.stefan@savba.sk (Š.N.); andrej.opalek@savba.sk (A.O.); 4 Centre of Polymer and Carbon Materials, Polish Academy of Sciences, M. Curie-Skłodowska 34, 41-800 Zabrze, Poland; jrydz@cmpw-pan.edu.pl; 5 Institute of Virology, Biomedical Research Center, Slovak Academy of Sciences, Dúbravská Cesta 9, 845 05 Bratislava, Slovakia; virumona@savba.sk

**Keywords:** electrospinning, therapeutic polymers, diclofenac sodium salt, poly(ε-caprolactone), biocompatibility, antibacterial activity

## Abstract

Diclofenac sodium salt (DSS)-loaded electrospun nanofiber mats on the base of poly(ε-caprolactone) (PCL) were investigated as biocompatible nanofibrous mats for medical applications with the ability to inhibit bacterial infections. The paper presents the characteristics of fibrous mats made by electrospinning and determines the effect of medicament on the fiber morphology, chemical, mechanical and thermal properties, as well as wettability. PCL and DSS-loaded PCL nanofibrous mats were characterized using scanning electron microscopy, transmission electron microscopy, attenuated total reflectance-Fourier transform infrared spectrometry, dynamic mechanical analysis, and contact angle measurements. Electron paramagnetic resonance measurements confirmed the lifetime of DSS before and after application of high voltage during the electrospinning process. In vitro biocompatibility was studied, and it was proved to be of good viability with ~92% of the diploid human cells culture line composed of lung fibroblast (MRC 5) after 48 h of incubation. Moreover, the significant activity of DSS-loaded nanofibers against cancer cells, Ca Ski and HeLa, was established as well. It was shown that 12.5% (m/V) is the minimal concentration for antibacterial activity when more than 99% of *Escherichia coli* (Gram-negative) and 99% of *Staphylococcus aureus* (Gram-positive) have been exterminated.

## 1. Introduction

Poly(ε-caprolactone) (PCL) shows excellent biocompatibility, resorbability, and high permeability to many drugs. Due to this fact, PCL is one of the most used synthetic polymers for medical applications, such as controlled drug delivery or release systems with a single bolus administration [1,2], as well as wound dressing [3,4] or regenerative tissue engineering [5,6]. Release systems, or drug delivery vehicles, have received increased attention in pharmaceutical treatment because they afford several advantages compared to conventional forms of dosage, including enhanced therapeutic efficacy and low toxicity when the drug is released at a controlled rate [7,8,9,10,11]. The release time of the drug needs to be prolonged for days or even several weeks. This may be provided by entrapping the biomolecules, such as drugs, proteins, DNA, or growth factors, within the polymer matrices. The authors already discussed the carriers of medicaments as particles [12,13,14], micelles [15,16,17], fibers [18,19,20], or polymer-drug conjugates [21,22,23]. 

Recently, electrospinning gained great popularity in producing drug-loaded electrospun fibers with diameters ranging from tens of nanometers to several micrometers. This method seems to be the most straightforward way to make fibers using a high voltage electrostatic field [24]. Electrospun materials have the benefit of unique properties, for instance, a large surface area to volume ratio, and enhanced cellular interactions [25]. The electrospun mats are attractive in medical applications due to their structure being similar to the extracellular matrix (ECM) [26,27,28,29,30,31]. 

The electrospun mats containing the drugs have multiple potential functions such as temporary support, drug delivery capabilities, and antibacterial properties to help prevent infections after, for example, surgery [14,32,33,34]. To prepare such type of therapeutics on the base of polymers, the simplest way is to mix the medicament with the polymer solution and incorporate it during electrospinning into the fibers’ structure. A well-known medicament, which is supplied under various trade names, is diclofenac sodium salt (DSS). DSS is a nonsteroidal drug (NSAID) of the phenylacetic acid class with anti-inflammatory, analgesic, and antipyretic properties.

Contrary to the action of many traditional NSAIDs, DSS inhibits cyclooxygenase COX-2 enzyme with greater potency than it does COX-113. Moreover, it has been shown to decrease the growth of pancreatic and non-small cell lung cancer xenografts and inhibit the growth of ovarian cancer cells [35,36,37,38,39]. Due to the benefits mentioned above, the diclofenac sodium salt was used as a model system in this study.

The present study is focused on the properties of the pharmaceutical drug diclofenac sodium salt incorporated into PCL nanofibers produced by electrospinning. The drug-loaded fibers were characterized by fundamental methods such as scanning electron microscopy (SEM), attenuated total reflectance-Fourier transform infrared spectroscopy (ATR-FTIR), mechanical properties studied by dynamic mechanical analysis (DMA), and thermogravimetric analysis (TGA). The wettability was assessed from the contact angle point of view. This study provides some evidence of DSS activity even after being subjected to high tension during the electrospinning process. The antibacterial activity against *Staphylococcus aureus*, Gram-positive bacteria (G+), and *Escherichia coli*, Gram-negative bacteria (G−) was studied. The minimal concentration of 12.5% (m/V) of DSS for antibacterial activity more than 99% was established, and in vitro biocompatibility of the human cells of the DSS-loaded electrospun PCL nanofibers were studied. Finally, the efficiency against HeLa and Ca Ski cancer cells was estimated. The prepared fibrous system exhibits potential for use as wound dressings with the ability to inhibit the infections caused by bacteria.

## 2. Materials and Methods

### 2.1. Materials

Biodegradable poly(ε-caprolactone) (PCL) CAPA 6800 from Solvay Interox Ltd. (Warrington, United Kingdom) with a mass-average molar mass of *M_w_* = 6.72 × 10^4^ g⋅mol^−1^ and a molar-mass dispersity of *Đ_M_* = 1.56 obtained by gel permeation chromatography (GPC) measurement (see Section 2.2.1), *N*,*N*-dimethylformamide (DMF) HPLC grade >99.7% (Alfa Aesar™, Karlsruhe, Germany), dichloromethane (DCM) anhydrous, ≥99.8%, containing 50–150 ppm amylene as a stabilizer from Sigma-Aldrich (Weinheim, Germany), absolute ethanol SOLVANAL, 99.8% (Centralchem, Bratislava, Slovakia), (2,2,6,6-tetramethyl piperidine-1-yl)oxyl (TEMPO•, Sigma-Aldrich, Poznań, Poland), diclofenac sodium salt (DSS), Phosphate Buffered Saline (PBS) and dimethyl sulfoxide (DMSO) from Sigma-Aldrich (Weinheim, Germany) were used for the experiments. Chloroform HPLC grade > 99.7% from Fisher Scientific UK Ltd. (Loughborough, UK) was used as a GPC eluent. The bacteria used in this study were *Staphylococcus aureus* (CCM 3953) and *Escherichia coli* (CCM 3988), purchased from the Czech Collection of Microorganisms, Masaryk University (Brno, Czech Republic). The strains were equivalent to ATCC 8739 and ATCC 6538P and were selected to comply with ISO 22196:2011 concerning the measurement of antibacterial activity on plastic surfaces. 3-(4,5-Dimethyldiazol-2-yl)-2,5-diphenyltetrazolium bromide (MTT) was purchased from Calbiochem (Merck Millipore, Darmstadt, Germany). Dulbecco’s modified Eagle medium (DMEM), fetal calf serum (FCS), streptomycin, penicillin, and *L*-glutamine were purchased from Gibco (Life Technologies, Grand Island, NY, USA). Human cell lines, MRC 5—normal lung fibroblasts, Ca Ski—epidermoid carcinoma derived from the metastatic site in the small intestine, and HeLa—cervix carcinoma, were obtained from the collection of the Institute of Virology BMC SAV (Bratislava, Slovakia).

### 2.2. Methods

#### 2.2.1. Gel Permeation Chromatography (GPC)

The GPC instrumentation contains a pumping system type P102 from Watrex (Prague, Czech Republic) and an evaporative light scattering detector (ELSD) model ELS-1000 from PL-Agilent Technologies (Stretton, UK). A TSHgel GMHHR-M GPC column from Tosoh Bioscience (King of Prussia, PA, USA) was used. The flow rate of the eluent was set to 1 mL⋅min^−1^. The concentration of PCL was 10 mg⋅mL^−1^ with chloroform as the eluent. The GPC measurements were performed at an ambient temperature. Molar masses are equivalent to PS calibration. Data were collected and processed with the help of Clarity software from DataApex (Prague, Czech Republic). 

#### 2.2.2. Preparation of Polymer Solution for Electrospinning

PCL solutions were prepared at a concentration of 10% (m/V) in the mixture of DCM/DMF solvents in the 1/1 volume ratio. PCL granules were weighted into the vials, and then the DCM was added. The resulting solution was intensively stirred on an IKA magnetic plate (Staufen im Breisgau, Germany) at an intensity of 750 rpm. After it was dissolved in DCM, the DMF was added to reach the required concentration. Diclofenac sodium salt was added to the polymer solutions in the concentrations listed in Table 1. The solutions were stirred until the DSS was dissolved. 

#### 2.2.3. Electrospinning (ESP)

Electrospinning was carried out under ambient temperature and humidity (24 °C ± 1 °C and 62% ± 1%, respectively) in a horizontal spinning configuration with a flat-end needle with a 0.8 mm (21 G) inner diameter. The working distance between the tip of the needle and the ground collector was 14 cm. The applied voltage was 15 kV, which was investigated by gradual experiments, with positive polarity. The voltage was driven by a high voltage power supply (Spellman SL-150W, Bochum, Germany). The solutions were fed through a single syringe pump model NE-1000 (New Era Pump Systems, Inc., Farmingdale, NY, USA). The feeding rate was 1.5 mL⋅h^−1^. The electrospun fibers were collected on aluminum foil. 

#### 2.2.4. Scanning Electron Microscopy (SEM)

Electrospun PCL-based fibers’ morphologies and the average diameter of fibers were observed by JSM Jeol 6610 scanning electron microscope (Tokyo, Japan) at an accelerated voltage of 10 kV. The samples were sputtered with a thin layer of gold. AzTec software (Springfield, NJ, USA) was used for collecting figures and processing the results. 

For SEM analysis of electrospun fibers after antibacterial testing, the procedure described by Bandeira et al. [40] was used. The samples were washed twice with a phosphate buffer solution (PBS, pH 7.4). Electrospun fibers were fixed with 4% paraformaldehyde for 30 min at room temperature. The samples were washed twice with PBS for 10 min and distilled water for 10 min. Subsequently, the samples were dehydrated with the addition of 25%, 50%, 70%, and 95% ethanol for 10 min and absolute ethanol twice for 15 min at room temperature.

#### 2.2.5. Transmission Electron Microscopy (TEM)

Topographical and morphological information was provided by JEOL 1200FX transmission electron microscope (Tokyo, Japan) operated at 80 kV. Samples were placed and observed on copper grids.

#### 2.2.6. Attenuated Total Reflectance-Fourier Transform Infrared Spectroscopy (ATR-FTIR)

Nicolet 8700 spectrophotometer (Thermo Fisher Scientific, Madison, WI, USA) was used for ATR-FTIR spectra recording, with a deuterated triglycine sulfate and thermoelectrically cooled (DTGS TEC) detector in the region of 600–4000 cm^−1^ with the resolution of 4 cm^−1^ using the absorbance mode.

#### 2.2.7. Thermogravimetric Analysis (TGA)

Thermal analysis of the investigated samples was performed with a Mettler-Toledo 851e thermogravimetric analyzer (Columbus, Ohio, USA). The measurements were carried out under constant nitrogen flow (50 mL⋅min^−1^) at a heating rate of 10 °C min^−1^ in the range of 24–500 °C. Approximately 1–3 mg of the sample was weighed and sealed in an aluminum pan. As a reference sample, the empty aluminum pans were used.

#### 2.2.8. Dynamic Mechanical Analysis (DMA)

The dynamic mechanical analysis of the different PCL fibers was performed on a DMA Q 800 machine (TA Instruments, New Castle, DE, USA) using the DMA controlled force procedure and stress/strain test method at a preload of 0.01 N and a force track of 1 N⋅min^−1^ up to an 18 N limit using a tensile setup. The rectangular shape of PCL samples with length 20 mm and width 5 mm was maintained at a constant temperature of 25 °C. The stress (MPa) and deformation (%) were recorded during the force track at 1 N⋅min^−1^ extension. 

#### 2.2.9. Measurements of Contact Angle

Static water contact angle measurements of all electrospun mats were performed at room temperature (24 °C ± 1 °C). Pure water droplets were used with a drop volume of 10 µL. For taking images, the Canon PowerShot SX130 camera (Tokyo, Japan) was used. The baseline at the surface of the nonwoven mats and the droplet interaction, and then the tangential line from the point of contact and the outer surface of the droplet, were drawn with the Image J software (LOCI, University of Wisconsin, WI, USA). The angles between these two lines were recorded as the contact angle.

#### 2.2.10. Electron Paramagnetic Resonance (EPR) Analysis

A Bruker WinEPR Processing device was used for the EPR analysis. A blank solution (50 μM) was prepared from 0.78 mg of TEMPO• in 10 mL DCM/DMF. The concentration of the investigated DSS was 1.48 mg⋅mL^−1^. The DSS solution after encapsulation into the fibers structure during electrospinning was prepared by releasing the DSS from the PCL/12.5%DSS mat. The sample with a thickness of < 1 mm and a mass of approx. 5 mg was put into the 4 mL phosphate buffer solution (PBS, pH 7.4) for 24 h for releasing. The EPR signals recording took 5 min after the start of the reaction. The parameters of the measurement were field modulation 100 kHz, modulation amplitude 1 G, field constant 40.96 ms, conversion time 671.089 ms, center field 3245 G, sweep width 100 G, X-band frequency 9.64 GHz, power 20 mW and temperature 25 °C.

#### 2.2.11. Antimicrobial Activity

The antimicrobial activity of the electrospun pure PCL and DSS-loaded PCL fibrous mats was determined with adherence to the procedure stipulated under ISO 22196:2011 for *Staphylococcus aureus* and *Escherichia coli* [41]. Bacterial suspensions were prepared at concentrations between 2.5 × 10^5^ and 10 × 10^5^ cells⋅mL^−1^. The 40 mm × 40 mm samples and square pieces of polyethylene film to cover them were placed under UV-light for 30 min to sterilize the material before the experiments. Afterward, 400 μL of the suspension was applied to the surface of the samples (pure PCL and DSS-loaded PCL mats). Samples with suspension were covered with the polyethylene film. After the contact time of 24 h, the samples (pure PCL and DSS-loaded PCL mats) were rinsed with 10 mL of casein–peptone lecithin polysorbate (CPLP) broth (base, Merck, Burlington, MA, USA) on a Petri dish, and the value for colony-forming cells (CFU/mL) was determined. The logarithm of reduction in the number of living and viable cells of tested bacteria (antibacterial activity, *R*) was calculated according to Equation (1):

*R* = (*U_t_* − *U*_0_) − (*A_t_* − *U*_0_) = *U_t_* − *A_t_* (1)

where *U*_0_ is the average value for the common logarithm of the number of viable bacteria [cells⋅cm^−2^], recovered from the control samples (pure PCL) immediately after inoculation, *U_t_* is the mean for the common logarithm of the number of viable bacteria, in [cells⋅cm^−2^], recovered from the control samples (pure PCL) after 24 h, *A_t_* is the mean for the common logarithm of the number of viable bacteria [cells⋅cm^−2^], recovered from the test samples (PCL with different concentrations of DSS) after 24 h. 

#### 2.2.12. Cell Viability Assay

A standard colorimetric MTT assay was used to assess the cytotoxicity of the prepared pure PCL and DSS-loaded PCL nanofiber mats (1 cm in diameter and ~1 mm thickness). The mats were UV-C sterilized for 30 min using germicidal lamp GT20T10, 20 W, 254 nm (Sankyo Denki, Japan) before incubation with cells. For the MTT assay, cells were seeded in 12-well tissue culture plates at a concentration of 1 × 10^5^ per well and incubated overnight in a full growth medium (DMEM, 10% FCS, penicillin, and streptomycin) in a CO_2_ incubator at 37 °C, 5% CO_2_, and saturated humidity. Before the incubation, 1.5 mL of a fresh culture medium was added into cells, and 1 PCL mat was placed into each well. The incubation and extraction of DSS were performed at 24 and 48 h, respectively. After 24, or 48 h of cell treatment, the pure PCL and DSS-loaded PCL mats were removed from treated cells, and the culture medium was replaced with a fresh culture medium containing 0.5 mg⋅mL^−1^ MTT and incubated for 2 h. The absorbance of the DMSO extract was measured using a Multiskan™ FC Microplate photometer (Thermo Fisher Scientific, Waltham, MA, USA) at 595 nm. All samples were done in quadruplicates (n = 4). 

#### 2.2.13. Statistical Data Analysis

Image J software (LOCI, University of Wisconsin, Madison, WI, USA) was utilized to measure the average diameter of the fibers in the mats. The average diameters of the fibers and their distributions were estimated statistically from at least 50 measured values on the 5 independently prepared samples to ensure accuracy.

The contact angle was assessed from at least 10 values. The measurements were carried out on the 5 independently prepared samples. The values of stress at break (σ) and elongation at break (ε) are the averages of the five measurements. Data are expressed as the mean ± standard deviation (SD). 

Antimicrobial test results are given as the means of 3 independent experiments ± SD. The differences between the given groups were tested for statistical significance using Student’s t-test (* *p* < 0.05; ** *p* < 0.01; *** *p* < 0.001).

The cell experiment results are presented as mean ± SD in quadruplicates (n = 4) from three independent experiments. A statistical analysis was performed using OriginPro 2016 software by one-way ANOVA with subsequent means comparison using the Turkey test.

## 3. Results

The development of biocompatible therapeutic agents, such as a wound dressing, is highly dependent on the characteristics of the drug carriers. In this study, the freestanding pure PCL and DSS-loaded PCL fibrous mats were fabricated by electrospinning at parameters listed in Table 1 (section Materials and Methods). Electrospun nanofibers were analyzed by scanning and transmission electron microscopy, attenuated total reflectance-Fourier transform infrared spectroscopy, thermogravimetric analysis, dynamic mechanical analysis, and electron paramagnetic resonance. The biocompatibility, cytotoxicity, and antimicrobial activity of the PCL and DSS-loaded PCL mats were also tested.

### 3.1. Morphology Evaluation of Electrospun Mats

**Figure 1:**
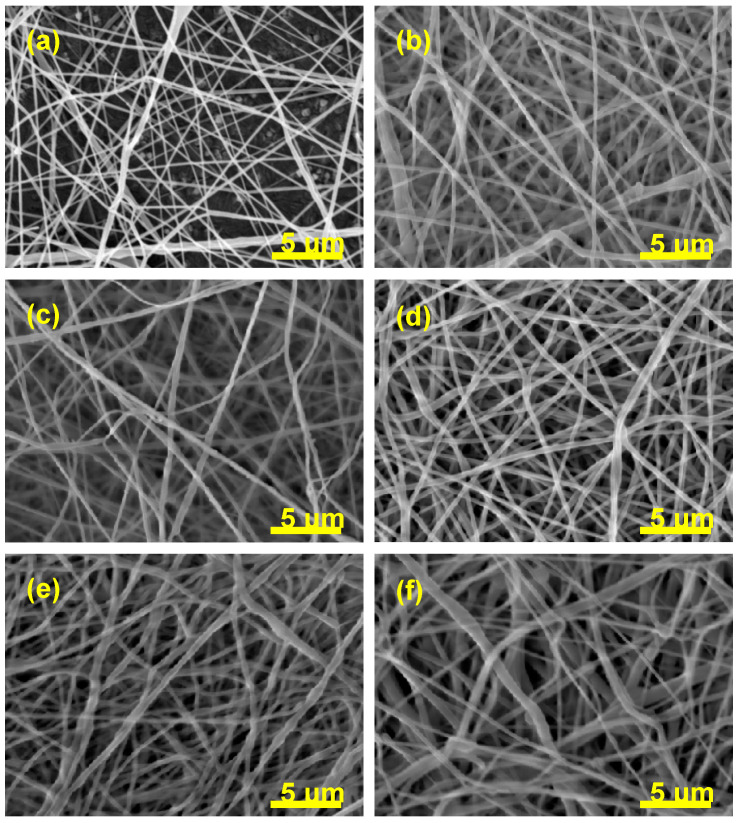
SEM micrographs comparing the fiber diameter and the distribution of diameters of pure poly(ε-caprolactone) (PCL) (**a**) and diclofenac sodium salt (DSS)-loaded PCL mats: PCL/2.5%DSS (**b**), PCL/6%DSS (**c**), PCL/12.5%DSS (**d**), PCL/25%DSS (**e**), and PCL/50%DSS (**f**) fibrous mats.

The morphology, as well as the fiber diameter and distribution of diameters of the electrospun fibers, has been investigated by scanning electron microscopy. The SEM micrographs are shown in Figure 1.

The values of diameters and their distribution are depicted in Table 2.

**Figure 2:**
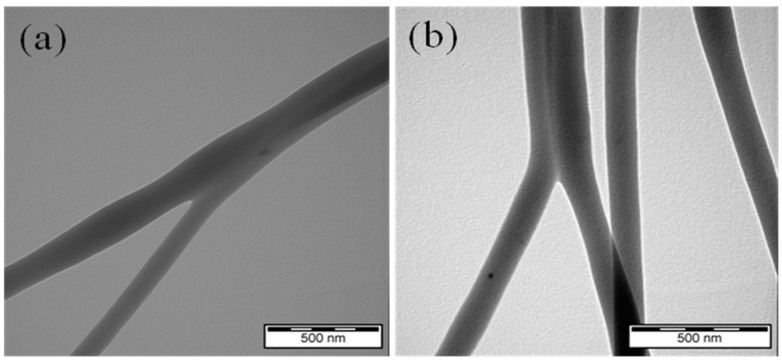
TEM micrograph of electrospun pure PCL (**a**) and PCL/125%DSS (**b**) fibrous mats.

The internal structure of the electrospun pure PCL and PCL/12.5%DSS fibrous mats were also analyzed using TEM (Figure 2).

### 3.2. ATR-FTIR Analysis of Pure PCL and DSS-Loaded PCL Fibrous Mats

**Figure 3:**
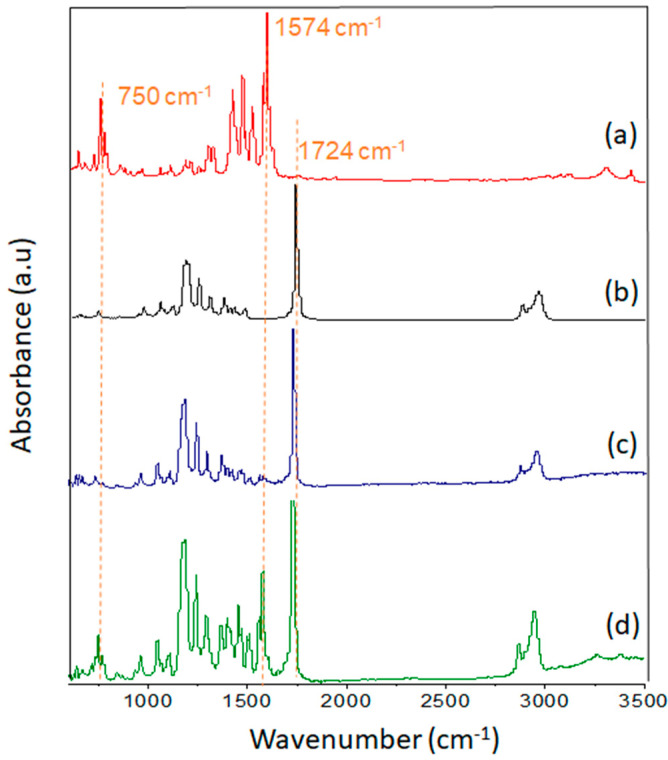
Infrared spectrum of diclofenac sodium salt (**a**), electrospun pure PCL (**b**), PCL/12.5%DSS (**c**), and PCL/25%DSS (**d**) fibrous mats.

ATR-FTIR analysis was employed to identify any changes in the chemical structure of the characterized material. ATR-FTIR gave clear proof of the DSS presence. With the increasing DSS concentration, the band at 1574 cm^−1^ appeared, and the intensity of the peak increased (Figure 3c,d).

### 3.3. Thermal Analysis of Pure PCL and DSS-Loaded PCL Fibrous Mats

**Figure 4:**
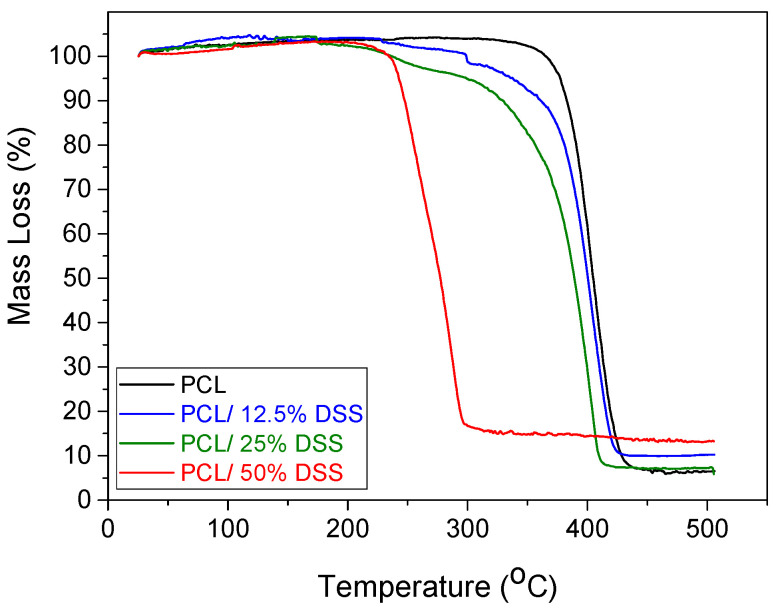
Thermogravimetric analysis of the studied samples: pure PCL (black line), PCL/12.5%DSS (blue line), PCL/25%DSS (green line) and PCL/50%DSS (red line) fibrous mats.

The TGA results of the electrospun pure PCL, PCL/12.5%DSS, PCL/25%DSS, and PCL/50%DSS fibrous mats are shown in Figure 4. The results indicate a decreased thermal stability with increasing DSS concentration.

### 3.4. Dynamic Mechanical Analysis of Pure PCL and DSS-Loaded PCL Fibrous Mats

It is important to determine the mechanical stability due to the need for manipulation and subsequent physiological forces [42]. Results of strain and stress of the PCL and DSS-loaded PCL mats performed by DMA are listed in Table 3.

### 3.5. Contact Angle Analysis of Electrospun Mats

**Figure 5:**
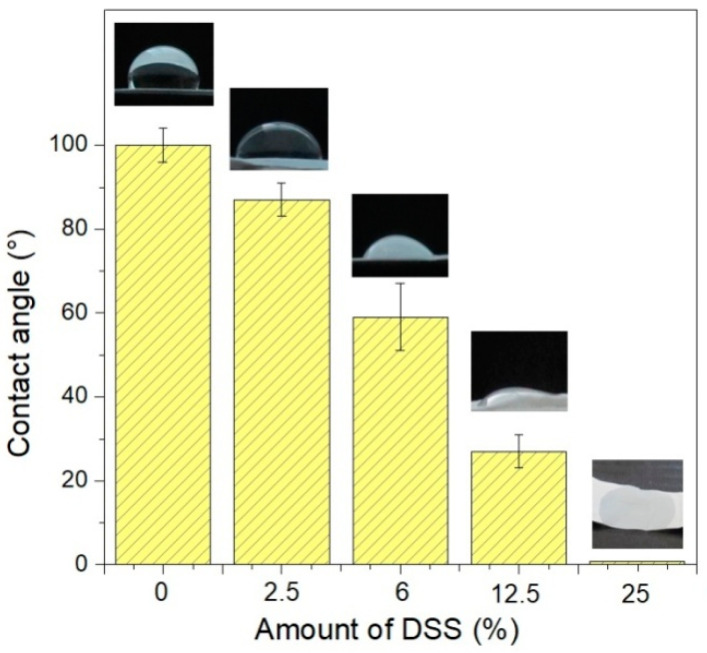
The water contact angle of the investigated samples: pure PCL, PCL/2.5%DSS, PCL/6%DSS, PCL/12.5%DSS, and PCL/25%DSS fibrous mats.

The water contact angle was measured to determine the wettability of pure PCL, PCL/2.5%DSS, PCL/6%DSS, PCL/12.5%DSS, PCL/25%DSS, and PCL/50%DSS mats. The water contact angle was evaluated depending on the DSS’s concentration in the PCL matrix, and the results are shown in Figure 5.

Table 4 shows the water contact angle values indicating the wettability of the electrospun mats. 

### 3.6. Electron Paramagnetic Resonance Analysis of Releasing DSS

**Figure 6:**
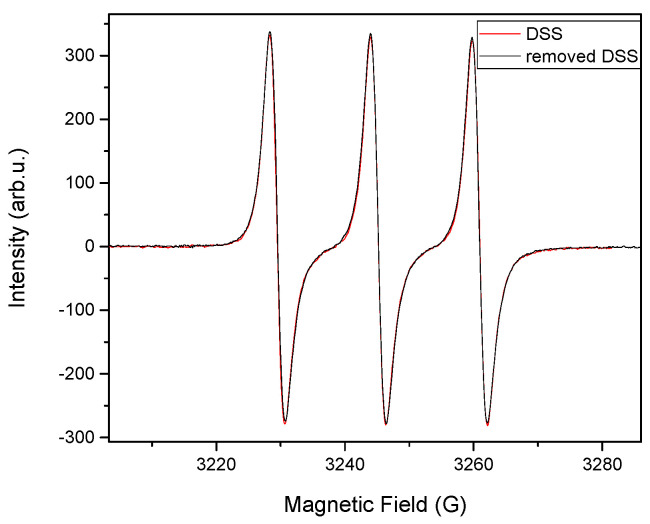
Electron paramagnetic resonance (EPR) spectra of removed DSS from PCL/12.5%DSS mat (black line) and the commercial DSS (red line) in DCM/DMF solution in the presence of TEMPO•.

EPR spectroscopy was used to study the radical scavenging activity of diclofenac sodium salt after incorporating electrospun mats using high voltage. The purpose was to determine the activity of DSS after using high voltage. The activity of the DSS releasing from PCL/12.5%DSS mats was compared to the original DSS activity that was not processed at high voltages. The results are listed in Figure 6.

### 3.7. Antimicrobial Activity Evaluation

**Figure 7:**
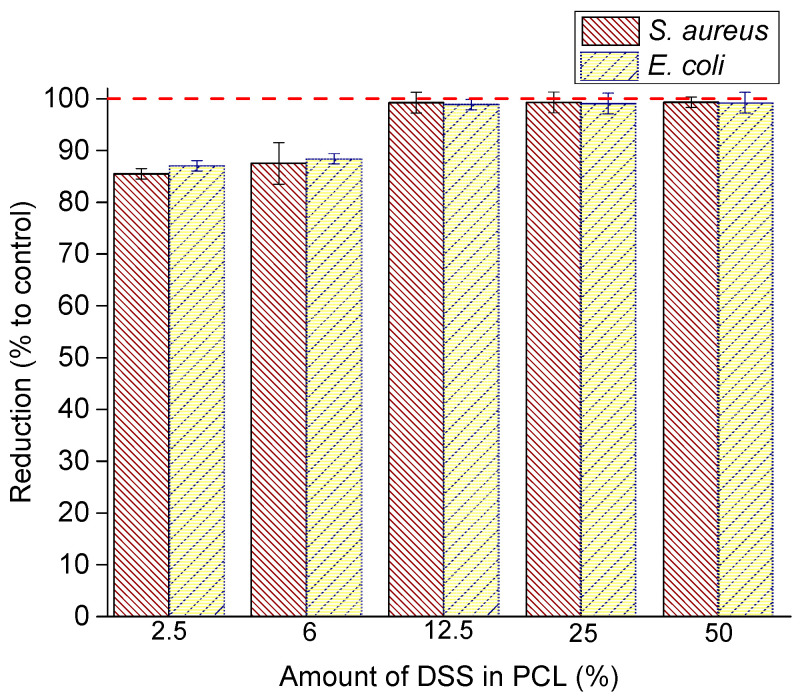
Antibacterial activity of DSS-loaded PCL mats: PCL/2.5%DSS, PCL/6%DSS, PCL/12.5%DSS, PCL/25%DSS, and PCL/50%DSS fibrous mats against *S. aureus* and *E. coli*.

Antimicrobial activity of electrospun pure PCL and DSS-loaded PCL fibrous mats: PCL/2.5%DSS, PCL/6%DSS, PCL/12.5%DSS, PCL/25%DSS, and PCL/50%DSS was determined with adherence to the procedure stipulated under ISO 22196:2011 for *Staphylococcus aureus* as Gram-positive and *Escherichia coli* as Gram-negative bacteria, separately. The bacterial viability of samples was investigated after 24 h. The reduction is graphically depicted in Figure 7.

The results of antimicrobial activity and reductions are listed in Table 5.

**Figure 8:**
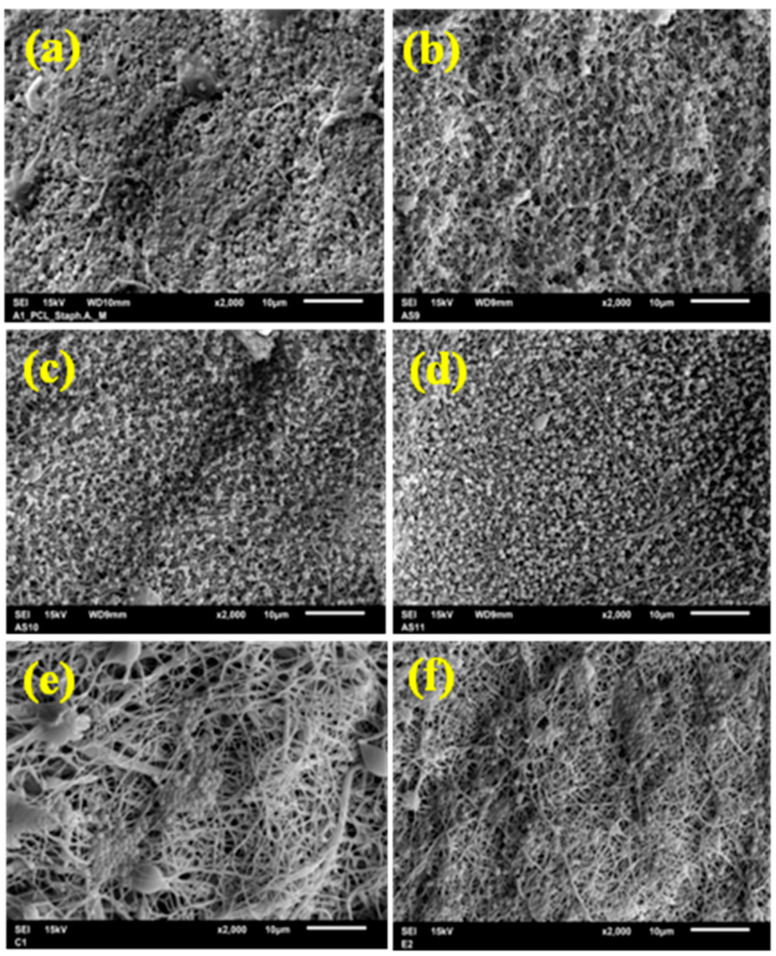
SEM micrograph showing the effect of the DSS concentration (wt%) on antibacterial efficacy against *S. aureus*: pure PCL (**a**), PCL/2.5%DSS (**b**), PCL/6%DSS (**c**), PCL/12.5%DSS (**d**), PCL/25%DSS (**e**), and PCL/50%DSS (**f**) fibrous mats.

**Figure 9:**
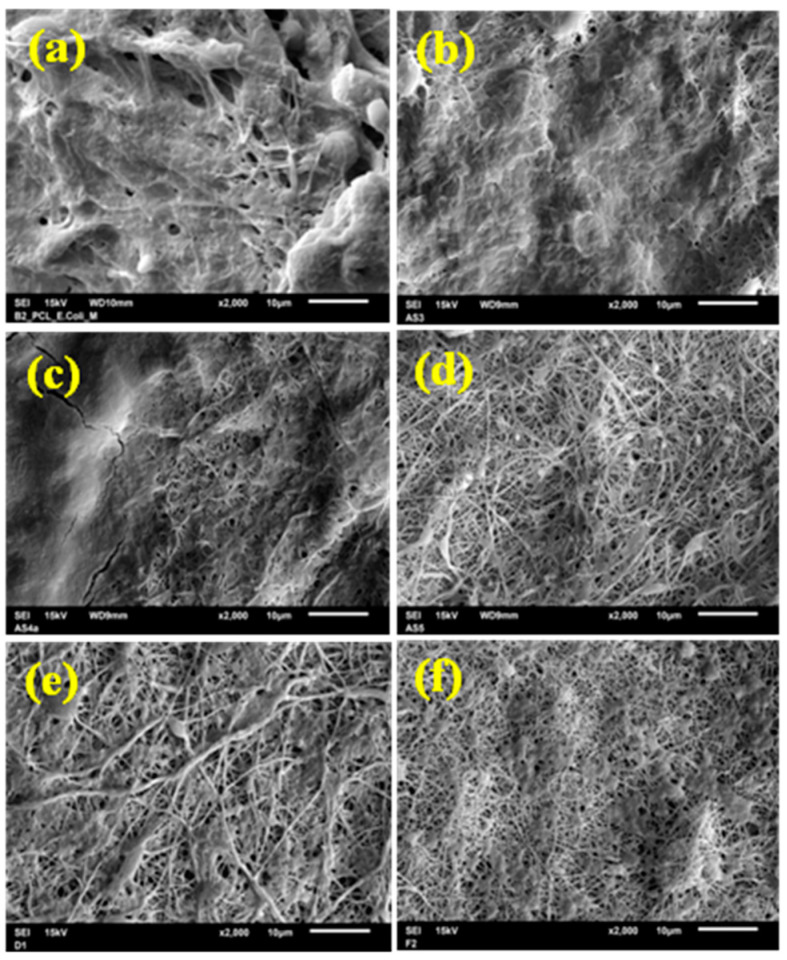
SEM micrograph showing the effect of the DSS concentration (wt%) on antibacterial efficacy against *E. coli*: pure PCL (**a**), PCL/2.5%DSS (**b**), PCL/6%DSS (**c**), PCL/12.5%DSS (**d**), PCL/25%DSS (**e**), and PCL/50%DSS (**f**) fibrous mats.

The improvement in antimicrobial efficiency with increasing DSS reduction is noticeable as a reducing bacterial viability (Table 5) and can be assessed optically from the SEM micrograph. SEM micrographs after 24 h incubation with *S. aureus* are shown in Figure 8. SEM micrographs after 24 h incubation with *E. coli* are shown in Figure 9.

### 3.8. Biocompatibility and Antiproliferative Effect of PCL and DSS-Loaded PCL Fibrous Mats

**Figure 10:**
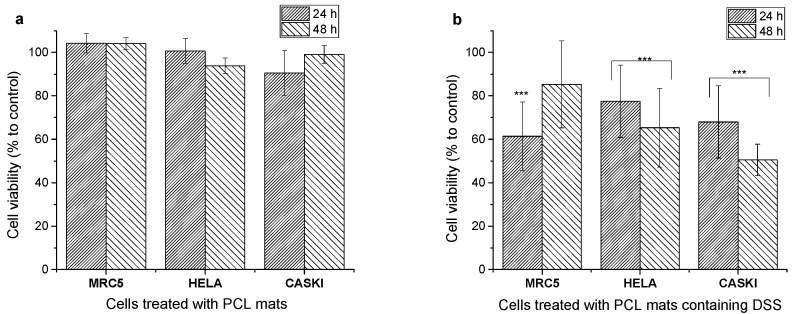
Biocompatibility of PCL mats (**a**) and anticancer activity of PCL mats loaded with DSS (**b**) after 24 h and 48 h incubation time, respectively. Cell viability was determined by MTT assay. The results are presented as mean ± SD (n = 12) of three independent experiments. The statistical significance is shown by asterisks (*** *p* < 0.001).

The PCL/12.5%DSS fibrous mat was selected for the evaluation of biocompatibility and the antiproliferative effect, as it showed relatively good mechanical properties and thermal stability with very good antimicrobial activity. The viability of MRC 5, HeLa, and Ca Ski cells was investigated to assess the biocompatibility of electrospun pure PCL and PCL/12.5%DSS fibrous mats after 24 and 48 h of incubation. The results are shown in Figure 10.

**Figure 11:**
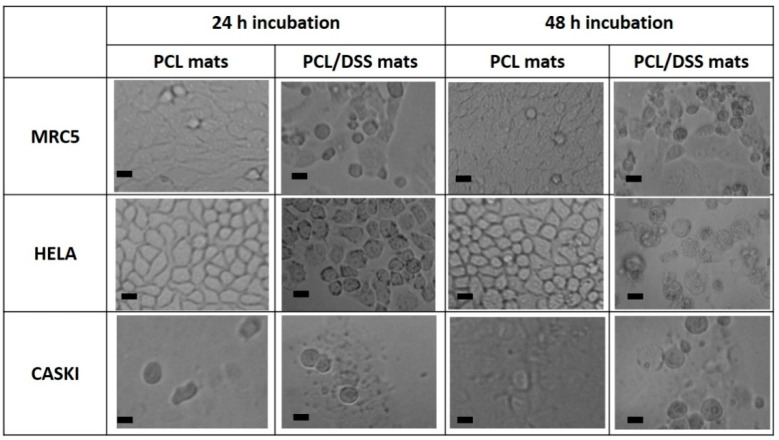
Microscopy images of cell line morphology. The scale bar corresponds to 20 μm.

The morphologies of MRC 5, HeLa, and Ca Ski cells in the presence of electrospun pure PCL and PCL/12.5%DSS mats were investigated as well. The morphology of cells after incubation times of 24 and 48 h is shown in Figure 11.

## 4. Discussion

Investigated pure PCL, as well as PCL/2.5%DSS, PCL/6%DSS, PCL/12.5%DSS, PCL/25%DSS, and PCL/50%DSS fibrous mats were fabricated by electrospinning from the mixture of DCM/DMF solvents under ambient temperature. The voltage of 15 kV and the feeding rate of 1.5 mL⋅h^−1^ were used to format beadles’ fibers. The freestanding mats were collected on alumina foil, from which they were removed before characterization and testing.

Two parameters have been identified that play an essential role in the electrospinning process: solute concentration and type of polymer used [43]. Therefore, through gradual experiments and literature studies [44,45,46], the proper concentrations required for fiber formation were optimized to avoid the beads, and obtain mats with good mechanical integrity. Appropriate selection and proportion of organic solvents, their dielectric properties, volatilities or boiling points are the basis for the formation of uniform fiber mats by electrospinning. In addition, smooth defect-free nanofibers with a narrow and more uniform diameter distribution are collected using two-component solvent systems. There have already been various solvents used, including benign or green, to prepare the PCL solutions for electrospinning, such as acetone, methanol, formic or acetic acid, or ionic liquids [47,48,49]. However, in these solvents it is not possible to achieve the required fiber characteristics (smoothness, small average diameter, uniform); therefore, in the conducted study, the solutions for electrospinning were prepared using a mixture with DCM as a good solvent for PCL. DCM is widely used in the pharmaceutical industry as a process solvent for which the Food and Drug Administration (FDA) has established residue tolerances [50]. Moreover, DMF was selected as co-solvent due to its suitable properties of a high dielectric constant, conductivity and lower vapor pressure [51]. DMF is also widely used in the pharmaceutical industry and due to its strong solvating properties and organic reactions occurred in it, which often cannot be achieved in less polar solvents, it is a unique solvent in chemical transformations [52].

The morphology, as well as the fiber diameter and distribution of diameters of the electrospun fibers, has been investigated by SEM. PCL electrospun fibers without beads were obtained from the mixture of the solvents consisting of DCM and DMF at a concentration of 10% (m/V). In medical applications, it is important that the fibers do not contain beads because the diameter of the fibers must mimic the natural extracellular morphology and thus promote optimal cell growth [2]. With the addition of DMF to the electrospinning solution, it is possible to transform the polymer particles into fibers with a smaller diameter [51]. The SEM micrograph of electrospun pure PCL shown in Figure 1a revealed that the diameter of PCL fibers in the nonwoven mat range from 78 nm to 500 nm with an average diameter of 162 nm ± 75 nm. This is significantly less than it has already been published [53,54,55] due to the balance of processing conditions as well. The individual electrospun fibers appear to be randomly distributed and generally had a similar thickness along with the fiber. About 50 fibers were analyzed to determine the diameter distribution.

The same experimental conditions were used to electrospin the samples with diclofenac sodium salt in various concentrations (see Materials and Methods section). The electrospun fibers were randomly arranged in the mats, as shown in Figure 1b–f, performed by SEM analysis. The low salt content improved spinnability (the formed fibers were thinner and uniform) as it increased the conductivity and decreased the surface tension of the solution. Overall, the addition of salt drastically affects the morphology and diameter of the resultant structures [56]. A high concentration of additives, such as salt or surfactants, into the polymer solutions could change their properties and causes the formation of beads or beads on the string [57]. Nevertheless, the experiments in this study showed that the electrospun fibrous mats are entirely without beads, even in the highest concentration of 50% DSS. Increasing the diclofenac sodium salt concentration did not result in the formation of beads. The average diameter and distribution of diameters were analyzed for all samples. It was observed that the average diameters and their distributions increase with the increasing concentration of the DSS. The most significant change in the saverage diameter was in the case of PCL/50%DSS mat and was 290 nm ± 145 nm. The data for all the investigated samples are summarized in Table 2.

The internal structure of pure PCL and PCL/12.5%DSS mats was analyzed by TEM. The TEM analysis allows a more detailed study of internal structure defects. The results are shown in Figure 2. There were no visible changes observed in the structure of the fibers and the heterogeneity after the addition of DSS compared to the PCL without a DSS additive, contrary to previous research on the internal structure of the electrospun silk nanofibers loaded with casein/DSS [58]. The current study confirmed the fibrous structure’s homogeneity, which indicates that DSS was loaded into the fibers as an amorphous polymorph. A polymorph refers to compounds that have the same chemical formula but different crystal structures. The solubility of the polymorphs does not differ. It is generally known that many organic molecules, including diclofenac, can be obtained in more than one crystalline form [59]. The results of the TEM analysis suggest considering amorphization as a factor slowing down the crystallization process of diclofenac salt. This leads to the conclusion that in the conducted study, diclofenac is incorporated into the structure of nanofibers mainly in the amorphous state. This phenomenon has already been described in published studies [60], and the amorphous state can explain TEM micrographs without changes in the internal structure.

The structural analysis was examined by ATR-FTIR. The absorbance spectra of the electrospun pure PCL, of the diclofenac sodium salt, and of the electrospun DSS-loaded PCL mats in two different concentrations of DSS, 12.5 and 25 wt%, which were analyzed in the 600–4000 cm^−1^ region to prove the presence of the DSS in the fibers, are shown in Figure 3. An ATR-FTIR analysis (Figure 3b) showed the presence of PCL based on its strong band corresponding to the carbonyl stretching mode around 1724 cm^−1^. The 2945 and 2865 cm^−1^ bands are specific for the asymmetric and symmetric CH_2_ stretching of PCL. The 1293 cm^−1^ region corresponds to C–O and C–C stretching in the crystalline phase of PCL. By adding DSS into the polymer solution, changes in the ATR-FTIR spectrum and the new absorption bands appearing between 3400 and 3200 cm^−1^ (Figure 3c,d), corresponding to the –NH group of DSS, can be clearly observed. Strong bands around 1574 cm^−1^ belong to the C=C bonding, and peaks in the absorption range 700–1300 cm^−1^ could be attributed to the C–Cl and C–N bonds of DSS. In the spectrum in Figure 3d, the absorption bands are more distinct due to the higher DSS concentration compared to the spectrum in Figure 3c. These observations agree with in-depth studies in the available literature [61,62].

The thermal behavior of the electrospun pure PCL and DSS-loaded PCL mats is depicted in Figure 4. The TGA curve of the electrospun pure PCL shows stability up to 350 °C. The total thermal decomposition of the investigated samples occurred at a temperature of 450 °C [63,64]. The mass loss of electrospun DSS-loaded PCL mats occurs in two steps. TGA curves of PCL/12.5%DSS and PCL/25%DSS fibrous mats show the first step of mass loss at 230 °C and the second step at 350 °C. The first temperature of thermal decomposition can be attributed to the diclofenac sodium salt [65]; the second temperature of thermal decomposition can be attributed to the PCL. The decomposition temperature of PCL/12.5%DSS and PCL/25%DSS mats slightly decreased with increasing DSS concentration. In the case of the PCL/50%DSS mat, the temperature of decomposition is 290 °C. The DSS affects the thermal stability of the electrospun pure PCL mat. A shift towards a lower temperature of decomposition value indicates a decrease in polymer stability. The PCL/2.5%DSS and PCL/6%DSS fibrous mats showed no differences compared to the electrospun pure PCL mat; therefore, they are not depicted in Figure 4. Nevertheless, since all materials are organic substances, it is unusual to observe that approximately 5–15% of the initial mass remains at 500 °C. This interesting thermal behavior of DSS has been pointed to in the already published studies [65,66].

The mechanical stress/strain testing was carried out to determine the tensile strength (*σ*) and strain (*ε*) of the electrospun fibrous mats and to assess the additives’ impact on the mechanical properties. Generally, the mechanical properties of electrospun mats are rather weak due to the flexibility of the polymer backbone. The macromolecules chains in the solution tend to be highly coiled and entangled in this state. After electrospinning, the chains remain coiled. Such a polymer chain conformation will result in fibers with molecular chains that can slip when exposed to a tensile force, resulting in poor mechanical properties [67]. Another reason for poor mechanical properties can be the random arrangement of individual fibers in the mats [68]. The results of stress/strain testing are shown in Table 3. The strength of the pure PCL was 7.5 MPa ± 1.2 MPa. It should be noticed that the strength of pure PCL in this study is slightly higher than in already published studies [69,70]. This would be explained by the smaller average diameters, which indicates the higher molecular orientation in the fibers. DMA studies showed that the mechanical properties worsened as the amount of the DSS in the fibrous structure increased. The strength decreased from 7.5 MPa ± 1.2 MPa for the PCL pure to 1.5 MPa ± 0.3 MPa for the PCL/50%DSS fibrous mat. The strain also decreased as the DSS content increased from 260 ± 74% for PCL pure to 41 ± 23 for PCL/50%DSS.

Measuring the water contact angle is an essential technique for determining the surface wettability of mats by a liquid. It is important for the various electrospun mat applications to control their interaction with water; for example, hydrophilic mats show better affinity to cells [71,72]. The results of the contact angle of the PCL and DSS-loaded PCL mats are shown graphically in Figure 5. The water contact angle was evaluated depending on the concentration of the DSS in the PCL structure. In pure PCL, the contact angle was 100° ± 4°, which indicates the low wettability of the mat. The increasing DSS concentration improved the hydrophilicity of the mats. The contact angles of all the investigated samples are listed in Table 4. The PCL/6%DSS and PCL/12.5%DSS mats were well wettable, which resulted from the contact angle of 68° ± 8° and 33° ± 3°, respectively. The drops immediately penetrated the surface of the mats in the cases of PCL mats with a DSS concentration of 25 wt% and higher; the wettability was complete. This result is due to the polar character of diclofenac sodium salt.

The radical scavenging activity of diclofenac sodium salt in bulk and after its release from the PCL electrospun mat into the phosphate buffer solution (PBS, pH 7.4) in 24 h of incubation was studied using electron paramagnetic resonance spectroscopy. Free radicals were detected using the stable free radical TEMPO• after removing the DSS from the PCL mats. The TEMPO• is reduced when it reacts with the diclofenac sodium salt donating hydrogen, and this reduction is recorded based on the corresponding inhibition of the EPR spectrum [73]. No significant changes were detected in the quartet resonance of TEMPO•, as can be seen in Figure 6. This result confirms the antioxidant activity of DSS also after undergoing the high applied voltage and releasing from electrospun PCL/12.5%DSS mat. The result is comparable with the previous study conducted on electrospun silk nanofibers loaded with casein/DSS [58].

The antimicrobial activity of diclofenac sodium salt was described by many authors [74,75]. In this study, the antimicrobial activity of the electrospun pure PCL and DSS-loaded PCL mats was tested against *S. aureus* and *E. coli* by the contact method according to ISO 22196:2011. The selected bacterial strains are the most common bacteria that cause soft tissue infections that delay wound healing [76,77]. *S. aureus* most often causes skin infections [78], while *E. coli* is considered the primary source of burn wound infections [79]. The results of the antibacterial activity test are listed in Table 5 and Figure 7. The number of viable bacteria recovered was established. The results of this experiment showed that bacterial growth on the PCL mats at different concentrations of diclofenac sodium salt was reduced for both strains tested. The 2.5 wt% of DSS is a sufficient concentration to achieve more than 85% reduction in bacteria growth of both tested strains after 24 h of contact. With the increase in DSS concentration, there is a further reduction in growth. PCL with 12.5–50 wt% diclofenac sodium salt concentrations resulted in a 99% reduction in *E. coli* and *S. aureus* cells. The inhibition of bacterial growth with the increasing DSS concentration was confirmed by SEM micrograph, as seen in Figure 8 (*S. aureus*) and Figure 9 (*E. coli*). Studies have shown that diclofenac sodium salt is an effective inhibitor of the growth of selected bacteria, although TEM micrographs indicated the amorphous state of DSS incorporated into the nanofiber structure during electrospinning. Literature data indicate that a change in the crystalline state may also result in a change in efficiency [60]. Evidently, the DSS did not lose its efficiency.

Biocompatibility of electrospun PCL fibrous mats combined with natural or synthetic polymers and their use in biomedical applications have been demonstrated in the literature [25,80,81]. As expected, the drug-free PCL mats used in this study have proven to be biocompatible with the HeLa and Ca Ski cells used (Figure 10a). It was shown previously that PCL/PEG nanofibers could control dipyridamole drugs [82]. Diclofenac sodium salt was used as the model drug in this study, as it was shown to have anti-inflammatory properties in addition to anticancer activity [37]. The PCL/12.5%DSS mat was selected to study the effect of the released drug on cancer cells (HeLa and Ca Ski) and normal skin cells (MRC 5) as it exhibited relatively good mechanical properties and thermal stability with very good antimicrobial activity. As shown in Figure 10b, all of the treated cells’ viabilities after 24 h incubation were significantly decreased compared to the control. However, after an additional 24 h of incubation (48 h in summary), the normal skin cells (MRC 5) recovered, and their viability increased. The viability of cancer cells decreased compared to control cells and cells after a 24 h treatment with PCL mats containing DSS. The decrease in cell viability can be assigned to the increased concentration of a released drug. Moreover, Ca Ski cells were more sensitive to released drugs with a viability of less than 50% compared to HeLa cells with 60% viability.

The morphology of cells in the presence of PCL mats did not change; all three types of cells exhibited a typical shape after either 24 or 48 h of incubation (Figure 11). In the presence of DSS-loaded PCL mats, the released DSS caused a decrease in cell viability which was observed when changing morphological patterns showed necrosis signs. The presence of necrotic cells is more pronounced in both cancer cell lines compared to normal cells. These results confirm that biocompatible PCL fibrous mats could be used for the controlled release of drugs, such as DSS.

## 5. Conclusions

Pure PCL and DSS-loaded PCL fibrous mats were fabricated by electrospinning, and comprehensively characterized. Randomly distributed beadles’ fibers with good surface homogeneity were obtained for pure PCL, as well as for all concentrations of DSS, with average diameters fibers in the range of 162–290 nm. The addition of DSS above 6 wt% increased the average diameters as DSS concentration increased, as well as decreased polymer stability and mechanical properties, especially for the PCL/50%DSS fibrous mat, whereas the increasing DSS concentration improved the hydrophilicity of the fibrous mats. The electrospun mats were tested for antimicrobial activity against *S. aureus* (G+) and *E. coli* (G−) bacteria. It was observed that the reduction in viability of 99.25% for *S. aureus* and 98.83% for *E. coli* cells occurred with the PCL/12.5%DSS mat. The reduction in viability of *S. aureus* and *E. coli* was almost 100% for the sample with the maximum concentration of DSS (PCL/50%DSS mat). Cytotoxicity and biocompatibility were tested with human normal lung fibroblasts cells (MRC 5). The activity of the drug released from electrospun mats was studied against epidermoid carcinoma from the metastatic site in the small intestine (Ca Ski) and cervix carcinoma (HeLa).

The obtained results indicate that electrospun DSS-loaded PCL mats exhibit potential in therapeutic applications as wound dressings with the ability to inhibit bacterial infections. In particular, the PCL/12.5%DSS fibrous mat showed relatively good mechanical properties and thermal stability with very good antimicrobial activity, biocompatibility and good anticancer activity.

## Figures and Tables

**Table 1:** Solutions prepared for electrospinning.

Sample	PCL (g)	DSS (m/V%)	DCM (mL)	DMF (mL)
PCL	1	-	5	5
PLC/2.5%DSS	1	2.5	5	5
PCL/6%DSS	1	6	5	5
PCL/12.5%DSS	1	12.5	5	5
PCL/25%DSS	1	25	5	5
PCL/50%DSS	1	50	5	5

**Table 2:** Values of average diameters (avg. dia.) of the tested fibers and distribution of average diameters in the PCL and PCL/DSS electrospun mats.

Content of DSS [wt%]	0	2.5	6	12.5	25	50
**Avg. Dia. ± SD [nm]**	162 ± 75	164 ± 59	171 ± 58	233 ± 84	266 ± 114	290 ± 145

**Table 3:** Values of stress and strain of the investigated samples.

Content of DSS [wt%]	*σ* ± SD_σ_ (MPa)	*ε* ± SD_ε_ (%)
0	7.5 ± 1.2	260 ± 74
2.5	7.2 ± 1.3	233 ± 59
6	7.0 ± 1.5	135 ± 22
12.5	5.5 ± 1.0	73 ± 5
25	4.3 ± 0.1	56 ± 4
50	1.5 ± 0.3	41 ± 23

*σ*—tensile strength, ***ε***—strain, SD—standard deviation.

**Table 4:** Contact angles values of the investigated samples.

**Content of DSS [wt%]**	0	2.5	6	12.5	25	50
**Contact Angle [°]**	100 ± 4	87 ± 4	68 ± 8	33 ± 3	n.d.	n.d.

n.d.—not determined since the droplet soaked into the sample immediately after contact with the measured surface when measuring the contact angle.

**Table 5:** Antibacterial activity and efficacy (quantitative) *Staphylococcus aureus* CCM 3953 and *Escherichia coli* CCM 3988. The concentration of bacteria in the test inoculum is 500,000 CFU/mL.

Tested Microorganism	Amount of DSS [wt%]	The Number of Bacteria Recovered at 24 h Contact Time [CFU/mL]	Logarithm of the Number of Bacteria Recovered at 24 h Contact Time [CFU/mL]	Antimicrobial Activity (*R*)	Reduction [%]
***S. aureus*** **CCM 3953** (**G+**)	0	3,000,000	6.30	-	-
	2.5	290,000	5.46	0.8	85.50 ± 0.01
	6	250,000	5.40	0.9	87.50 ± 0.04
	12.5	15,000	4.18	2.1	99.25 ± 0.02
	25	14,500	4.16	2.1	99.28 ± 0.02
	50	14,000	4.15	2.2	99.30 ± 0.01
***E. coli*** **CCM 3988** (**G−**)	0	3,000,000	6.48	-	-
	2.5	390,000	5.59	0.9	87.00 ± 0.01
	6	350,000	5.54	0.9	88.33 ± 0.01
	12.5	35,000	4.54	1.9	98.83 ± 0.01
	25	29,500	4.47	2.0	99.02 ± 0.02
	50	24,000	4.38	2.1	99.20 ± 0.02

CFU—colony-forming cells, G+—Gram-positive bacteria, G−—Gram-negative bacteria.
